# Nystrom technique analysis of the terahertz and infrared range radar cross section of a circular dielectric cylinder with graphene strips inside

**DOI:** 10.1098/rsta.2024.0337

**Published:** 2025-08-14

**Authors:** Mstyslav E. Kaliberda, Sergey Pogarsky

**Affiliations:** ^1^ V N Karazin Kharkiv National University, Kharkiv, Ukraine

**Keywords:** radar cross section, absorption, graphene, integral equation, Nystrom-type algorithm

## Abstract

We study the modification of the radar cross section (RCS) of a circular dielectric cylinder with coplanar graphene strips inside, focusing on the variation of the chemical potential and the excitation of associated plasmon resonances. The scattering and absorption of the H-polarized plane wave in the THz and infrared frequency range, up to 55 THz, are considered. The mathematically grounded method of hyper-singular integral equations and the meshless Nystrom-type algorithm are used. The reduction of RCS is essential in stealth technologies. At the same time, the increase of RCS with the help of radar enhancers is also important and used in marine and air safety applications, making small boats, coastal structures or aircraft more visible for the radar. We show that RCS of the studied scatterer is tunable and can be both increased and reduced at the same frequency.

This article is part of the theme issue ‘Analytically grounded full-wave methods for advances in computational electromagnetics’.

## 1. Introduction

The reduction of radar cross section (RCS) is essential in stealth technologies. At the same time, the increase of RCS with the help of radar enhancers is also important in marine and air safety, making small boats, coastal structures and aircraft more visible to radar. The tunability of RCS is a highly desirable property.

Graphene is a relatively new material whose electrodynamic properties can be tuned by the application of electrostatic or magnetostatic doping [1–4]. The conductivity of graphene $\sigma =\sigma \left(f,{µ}_{c},\tau ,T\right)$ is a function of the frequency f, chemical potential ${µ}_{c}$, electron relaxation time τ and temperature T. Importantly, the chemical potential ${µ}_{c}$ can be controlled dynamically. Graphene exhibits remarkable strength and the ability to absorb electromagnetic fields. Additionally, it can support surface plasmon–polariton waves. Graphene strips can support plasmon natural waves and corresponding plasmon resonances at THz and infrared frequencies [5,6]. These characteristics make graphene attractive as an element of tunable antennas, sensors, absorbers and other devices [7–13].

Ideally, a single layer of graphene is only one atom thick. If the width of a graphene strip is greater than 100 nm, it can be considered as a zero-thickness resistive surface with complex-valued conductivity [14–16]. The conductivity of graphene can be obtained from the Kubo formalism [17,18].

In [19–31], the scattering and absorption by resistive strips or gratings of such strips are considered using the method of analytical regularization and the methods of integral equations. For discretization of the boundary integral equations, the Nystrom-type algorithms are used [30–32]. Unlike commercial packages, these methods provide controllable accuracy and guaranteed convergence.

The scattering by a dielectric cylinder covered with perfectly electric conducting strips is considered in several papers (see, for example [33–35]). The use of graphene strips to cover a dielectric cylinder is examined in [36,37] with the help of the method of integral equations. This configuration is proposed for controlling RCS and as an antenna element. In [38], dielectric cylinders fully covered with graphene are studied for the positioning of electron beams. Additionally, they are considered in [39] as tunable resonant elements in lasers when they include an active zone. The dielectric cylinder with an active zone and a single silver [40] or graphene strip [41] placed inside it is also examined as a laser element. The method of integral equations with Nystrom-type discretization is used. In [42], only basic equations for a laser based on a dielectric cylinder symmetrically loaded with a graphene strip are presented, without any numerical results or discussions of the physical phenomena. While these studies focus on single-strip configurations, the introduction of multiple graphene strips can change the scattering behaviour and enhance the system’s performance. In the case of multiple graphene strips, the localized plasmon modes of individual strips can couple with one another, leading to the formation of super-modes. The presence of super-modes often results in shifts in the resonance frequencies compared with the resonances of a single strip. By varying parameters such as the distance between strips or the chemical potential of graphene, it is possible to control the super-mode behaviour [38]. Super-modes can lead to a broader resonance bandwidth and stronger field enhancements compared with a single strip, which is particularly useful in applications where RCS enhancement is desired.

In this article, we consider the *H*-polarized (magnetic vector is parallel to the axis of the cylinder) plane wave scattering by a finite number of planar graphene strips placed inside the dielectric cylinder. We reduce the problem to the hyper-singular integral equations. The Nystrom-type discretization algorithm is full-wave and meshless. Its convergence is based on corresponding theorems about the approximation of singular and regular integrals using the quadrature rules. The H-polarization is considered, since here the plasmon resonances can arise.

## 2. Problem statement

**Figure 1.**
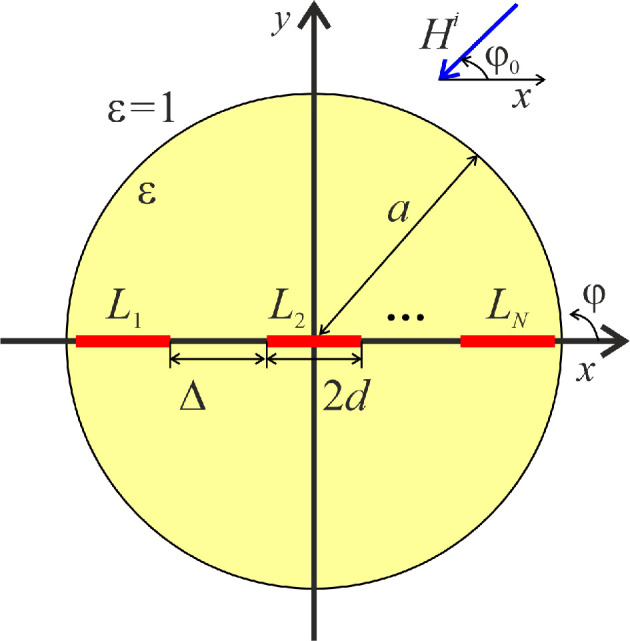
Structure geometry.

Let us consider a lossy dielectric cylinder of radius a and permittivity $\varepsilon_{0}\;\varepsilon$, where $\varepsilon_{0}$ is vacuum permittivity. The losses are introduced with the help of the imaginary part of ε, $\sqrt{\varepsilon }=\alpha +i\beta$, α>0, β>0. The axis of the cylinder coincides with the z-axis. A finite number of graphene strips is placed on the plane y=0 inside the cylinder (see figure 1). We denote the set of strips as $L=\overset{N}{\underset{n=1}{⋃}}L_{n}$, where $L_{n}$ is the nth strip, N is the number of strips. The time dependence is assumed to be exp(−iωt).

Suppose that the plane wave with unit amplitude is incident from the domain z>0,


(2.1) $$H_{z}^{i}\left(x,y\right)=exp\left(-ik\left(x\;cos\;\phi_{0}+y\;sin\;\phi_{0}\right)\right),$$

where k=2π/λ is the wave number in free space, $\phi_{0}$ is the incidence angle. The total field we seek is a sum of the primary field and scattered field, $H_{z}=H_{z}^{p}+H_{z}^{s}$. The primary field is the field in the absence of graphene strips.

We consider the following boundary-value problem. The fields should satisfy the Helmholtz equation with wave numbers k and $k_{1}$ (here $k_{1}=k\sqrt{\varepsilon }$ is the wave number in the dielectric), the boundary conditions at the vacuum–dielectric interface


(2.2) $$H_{z}(r\to a+0)=H_{z}(r\to a-0),$$

(2.3) $$\begin{array}{l} E_{\tau }\left(r\to a+0\right)=E_{\tau }\left(r\to a-0\right), \end{array}$$

where $r=\sqrt{x^{2}+y^{2}}$ is the polar distance, the boundary conditions on the graphene strips and outside the strips


(2.4) $$\begin{array}{c} \;E_{x}(y\to +0)=\frac{1}{\sigma }\left(H_{z}(y\to +0)-H_{z}(y\to -0)\right),x\in L, \end{array}$$

(2.5) $$\begin{array}{c} E_{x}(y\to +0)=E_{x}(y\to -0),x\in R, \end{array}$$

as well as the radiation and the edge conditions. The solution of the problem is unique.

## 3. Solution of the problem

To find the primary field, we represent it as a sum of cylindrical waves in each domain with unknown coefficients,


(3.1) $$H_{z}^{p}=\left\{ \begin{array}{ll} H^{i}+\sum_{m=-\infty }^{\infty }A_{m}H_{m}(kr)\exp\left(im(\phi -\phi_{0})\right), & r>a,\\ \sum_{m=-\infty }^{\infty }B_{m}J_{m}(k_{1}r)\exp\left(im(\phi -\phi_{0})\right), & r<a, \end{array}\right.$$

where ϕ is the polar angle, $H_{m}(x)$, $J_{m}(x)$ are the Hankel (first kind) and the Bessel functions. We also represent the incident plane wave as a sum of cylindrical waves with the help of the Fourier–Bessel expansion, $H_{z}^{i}=\sum_{m=-\infty }^{\infty }{\left(-i\right)}^{m}J_{m}\left(kr\right)exp\left(im\left(\phi -\phi_{0}\right)\right)$. From (2.2) and (2.3) coefficients


(3.2) $$A_{m}={\left(-i\right)}^{m}\frac{J_{m}^{\prime }\left(k_{1}a\right)J_{m}\left(ka\right)-\sqrt{\varepsilon }J_{m}\left(k_{1}a\right)J_{m}^{\prime }\left(ka\right)}{\sqrt{\varepsilon }J_{m}\left(k_{1}a\right)H_{m}^{\prime }\left(ka\right)-H_{m}\left(ka\right)J_{m}^{\prime }\left(k_{1}a\right)}$$

and


(3.3) $$B_{m}={\left(-i\right)}^{m}\sqrt{\varepsilon }\frac{H_{m}^{\prime }\left(ka\right)J_{m}\left(ka\right)-J_{m}\left(ka\right)J_{m}^{\prime }\left(ka\right)}{J_{m}\left(k_{1}a\right)H_{m}^{\prime }\left(ka\right)\sqrt{\varepsilon }-H_{m}\left(ka\right)J_{m}^{\prime }\left(k_{1}a\right)}$$

are obtained. We seek the scattered field as a sum of the double-layer potentials [40]:


(3.4) $$H_{z}^{sc}(\vec{r})=\sum_{n=1}^{N}\int_{L_{n}}w_{n}\left({\vec{r}}_{1}\right)\frac{\partial G\left(\vec{r},{\vec{r}}_{1}\right)}{\partial y_{1}}d{\vec{r}}_{1},y_{1}=0,$$

where $G\left(\vec{r},{\vec{r}}_{1}\right)$ is the Green’s function, $w_{n}\left({\vec{r}}_{1}\right)$ are unknown current densities on the strips, $\vec{r}=\left(x,\;\;y\right)$ and ${\vec{r}}_{1}=\left(x_{1},\;\;y_{1}\right)$ are radius-vectors of the observation point and the point on the strips. The Green’s function corresponds to the magnetic field produced by an infinite magnetic line source of unit strength positioned inside the dielectric cylinder. By integrating over the set of graphene strips, we ensure that the surface currents are confined to the strips, effectively imposing that the currents vanish outside the strip regions.

Here, in fact, we have two scattering geometries. One of them is the dielectric cylinder. Another one is the graphene strip grating. The Green’s function is a sum of two functions, $G=G_{0}+G_{1}$. Function $G_{0}$ is the two-dimensional free-space Green’s function, which is connected with the scattering by the strips in the infinite dielectric medium. Function $G_{1}$ accounts for the presence of the dielectric cylinder. Taking into account that considered scattering geometry consists of two domains—the inner and outer domains of the dielectric cylinder, *r* ≷ *a*—we obtain


(3.5) $$\begin{array}{l} G_{0}\left(\vec{r},{\vec{r}}_{1}\right)=\left\{ \begin{array}{ll} \frac{i}{4}H_{0}\left(k_{1}\left|\vec{r}-{\vec{r}}_{1}\right|\right), & r<a,\\ 0,r>a, \end{array}\right. \end{array}$$

(3.6) $$G_{1}(\vec{r},\vec{r_{1}})=\frac{i}{8}\sum_{m=-\infty }^{\infty }\;C_{m}J_{m}(k_{1}r)J_{m}(k_{1}r_{1})(exp(im\phi )-exp(-im\phi ))exp(im\phi_{1}),r<a$$

and


(3.7) $$G_{1}(\vec{r},\vec{r_{1}})=\frac{i}{8}\sum_{m=-\infty }^{\infty }\;D_{m}H_{m}(kr)J_{m}(kr_{1})(exp(im\phi )-exp(-im\phi ))exp(im\phi_{1}),r>a,$$

where


(3.8)Cm=(Hm′(k1a)Hm(ka)/ε−Hm′(ka)Hm(k1a))/αm,(3.9)Dm=2i/(πk1aαmε)

and


(3.10) $$\begin{array}{r} \alpha_{m}=H_{m}^{\prime }\left(ka\right)J_{m}\left(k_{1}a\right)-J_{m}^{\prime }\left(k_{1}a\right)H_{m}\left(ka\right)/\sqrt{\varepsilon } \end{array}.$$

In (3.6) and (3.7), only terms that are odd with respect to the angle ϕ are considered, while the even terms are neglected. This approach is based on the fact that the electric field vanishes for even terms at $\phi =0^{0}$ and the graphene strips are assumed to have zero thickness. Additionally, taking into account that $\frac{\partial G\left(\vec{r},{\vec{r}}_{1}\right)}{\partial y_{1}}=0$ for all x∉L, if $y=y_{1}=0$, the magnetic field of zero-thickness strips vanishes at y=0 off the strips. Hence, this field representation is mathematically consistent and correct for the scenario considered in the article.

The field in the form of (3.4), taking into account (3.5)–(3.10), satisfies the boundary conditions (2.2), (2.3), (2.5) and the radiation condition. From the edge condition, it follows that the function $w_{n}(x)$ approaches zero as a square root near the edges of the strips, $w_{n}(x)\sim \sqrt{\left|x-\theta \right|}$, if x→θ, where θ represents one of the edges of the strip.

The enforcement of (2.4), taking into account the limit value of the double-layer potential, gives the hyper-singular integral equation:


(3.11) $$\begin{array}{l} \frac{4k_{1}\sqrt{\varepsilon }}{\sigma Z_{0}}w_{q}\left(x\right)+k_{1}\sum_{n=1}^{N}\int_{L_{n}}^{}\frac{H_{1}\left(k_{1}|x-x_{1}|\right)}{|x-x_{1}|}w_{n}\left(x_{1}\right)dx_{1}\\ +\sum_{n=1}^{N}\int_{L_{n}}^{}\sum_{m=-\infty }^{\infty }C_{m}m^{2}sgn\left(x^{m-1}x_{1}^{m-1}\right)J_{m}\left(k_{1}|x|\right)J_{m}\left(k_{1}|x_{1}|\right)/|xx_{1}|w_{n}\left(x_{1}\right)dx_{1}\\ =4i\frac{\partial H_{z}^{p}\left(x,y=0\right)}{\partial y},x\in L_{q},q=1,...,N, \end{array}$$

where $Z_{0}=120\pi \Omega$ is the free-space impedance. The second term in (3.11) is the hyper-singular integral, which should be understood in the sense of the Hadamar finite part. Its integrand has a singularity of the form $1/|x-x_{1}{|}^{2}$, if $x_{1}\to x$.

Following [30,40,41], for the discretization of (3.11) using the Nystrom-type algorithm, we first change the variables to reduce the integration over $L_{n}$ to the integration over the standard interval [−1,   1], where $x_{1}\to t\in [-1,\;\;\;1]$, $x\to t_{0}\in [-1,\;\;\;1]$, and $w_{n}(x)\to W_{n}(t_{0})\sqrt{1-t_{0}^{2}}$. Considering that the principal term of the asymptotic expansion of the Hankel function behaves as $H_{1}(\tau )/\tau \sim (i/\pi )\ln\tau -2i/(\pi \tau^{2})$, τ→0, the left-hand side of (3.11) can be represented as a sum of three components: the Hadamar finite part integral with the singularity of the form $1/{\left(t-t_{0}\right)}^{2}$, an integral with the logarithmic singularity of the form $\ln|t-t_{0}|$, and a regular integral without singularities. Next, we substitute the unknown functions $W_{n}(t)$ with the Lagrange interpolation polynomial $P_{M-1}(t)$ of order M−1. To derive the resulting matrix equation, we apply the Gauss–Chebyshev quadrature rule with the Chebyshev weight $\sqrt{1-t^{2}}$. As nodes and collocation points, the zeros of the Chebyshev polynomials of the second kind on every strip are taken, given by $t_{j}=\cos(\pi \;j/M)$, j=1,...,M, where M is the number of nodes on every strip:


(3.12) $$\begin{array}{l} \frac{1}{\pi }\int_{-1}^{1}\frac{P_{M-1}\left(t\right)}{{\left(t-t_{0}\right)}^{2}}\sqrt{1-t^{2}}dt\\ =\frac{1}{M+1}\sum_{\begin{array}{c} j=1\\ j\neq s \end{array}}^{M}P_{M-1}\left(t_{j}\right)\frac{\left(1-t_{j}^{2}\right)\left(1-{\left(-1\right)}^{j+s}\right)}{{\left(t_{j}-t_{0}\right)}^{2}}-\left(M+1\right)\frac{P_{M-1}\left(t_{0}\right)}{2}, \end{array}$$

(3.13) $$\begin{array}{l} \;\frac{1}{\pi }\int_{-1}^{1}P_{M-1}\left(t\right)ln|t-t_{0}|\sqrt{1-t^{2}}dt\\ \;=\frac{1}{M+1}\sum_{j=1}^{M}P_{M-1}\left(t_{j}\right)\left(t_{j}^{2}-1\right)\\ \;\times \left(ln2+\sum_{m=1}^{M}\frac{2}{m}cos\frac{\pi \cdot m\cdot j}{M+1}cos\left(m\;arccost_{0}\right)+\frac{{\left(-1\right)}^{j}}{M+1}cos\left(\left(M+1\right)arccos\left(t_{0}\right)\right)\right), \end{array}$$

(3.14) $$\frac{1}{\pi }\int_{-1}^{1}P_{M-1}\left(t\right)\sqrt{1-t^{2}}dt=\frac{1}{M+1}\sum_{j=1}^{M}P_{M-1}\left(t_{j}\right)\left(1-t_{j}^{2}\right).$$

In (3.12)–(3.14), we assume that $t_{0}=t_{s}$, s=1,...,M, coincides with one of the collocation points.

## 4. Results of the numerical analysis

To study scattering and absorption, the figures of merit are the total scattering cross section (TSCS) and the absorption cross section (ACS). The power that is reflected back to the source can be described with the help of RCS. Taking into account the asymptotic representation of the Hankel function, the radiation pattern is


(4.1) $$\begin{array}{l} D\left(\phi \right)=\frac{1}{4}\sum_{n=1}^{N}\int_{L_{n}}^{}\sum_{m=-\infty }^{\infty }C_{m}m\;sgn\left(x_{1}^{m-1}\right)J_{m}\left(k_{1}|x_{1}|\right)/|x_{1}|\\ \times w_{n}\left(x_{1}\right)\left(exp\left(im\phi \right)-exp\left(-im\phi \right)\right)dx_{1}\\ +\sum_{m=-\infty }^{\infty }A_{m}exp\left(im\left(\phi -\phi_{0}\right)\right) \end{array}$$

and


(4.2)TSCS=2πk∫02π|D(ϕ)|2dϕ,(4.3)ACS=Re1σZ0⋅∑n=1N∫Ln|wn(x1)|2dx1,(4.4)RCS=4k|D(ϕ0)|2.

The power conservation law for the lossless dielectric is


(4.5) $$ACS+TSCS=-\frac{4}{k}ReD\left(\phi_{0}+\pi \right).$$

For the numerical results, we assume that identical strips are placed equidistantly. The strip width is 2d, the distance between two adjacent strips is Δ. The structure is symmetric with respect to the y-axis.

To study the numerical convergence, we introduce the relative error as follows:


(4.6) ξ(M)=|RCS(2M)−RCS(M)|/RCS(M).

**Figure 2.**
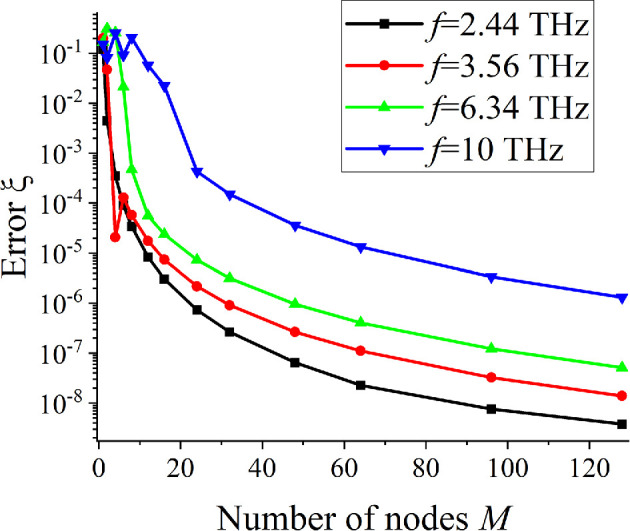
Dependences of the error on the number of nodes for a single graphene strip, N=1, ${µ}_{c}=1$ eV, d=10µm, a=50µm, ε=2.25, $\phi_{0}=90^{0}$.

Figure 2 shows dependences of the error on the number of nodes M. Since the convergence is theoretically guaranteed by the theorems, figure 2 allows us to determine its actual rate. The values of parameters are taken near the local maxima of RCS dependences on the frequency (presented below). Starting from a certain value of $M>M_{0}$, the convergence becomes monotonic. As the relative strip width kd increases, more interpolation nodes are required.

**Figure 3.**
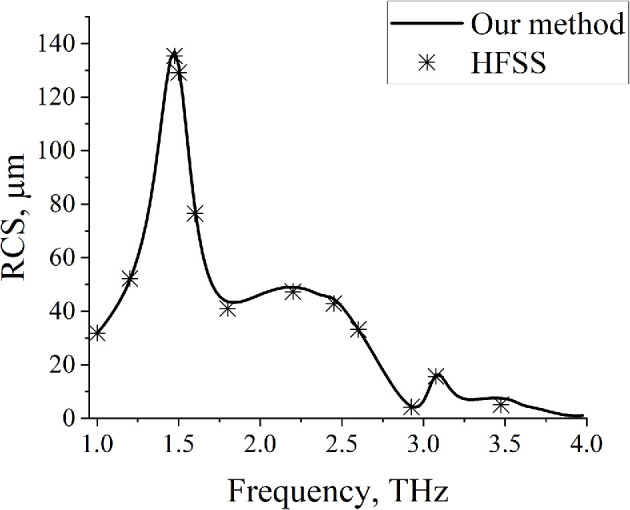
Dependences of RCS on the frequency. Comparison of our method (solid line) with HFSS (asterisks), N=2, d=10µm, Δ=20µm, a=40µm, ε=2.25, $\phi_{0}=90^{0}$.

To validate our results, we compare them with those obtained in HFSS (ANSYS EM Suite). The frequency dependences of RCS for N=2 strips, obtained by our method and in HFSS, are presented in figure 3. Good agreement is observed. Our homemade code allows for results to be obtained dozens of times faster.

We study the scattering and absorption of the graphene strips inside the dielectric cylinder in two frequency bands: 0.1–10 THz and 5–55 THz. In each frequency band, the width of the graphene strips is selected so that plasmon resonances can arise.

**Figure 4.**
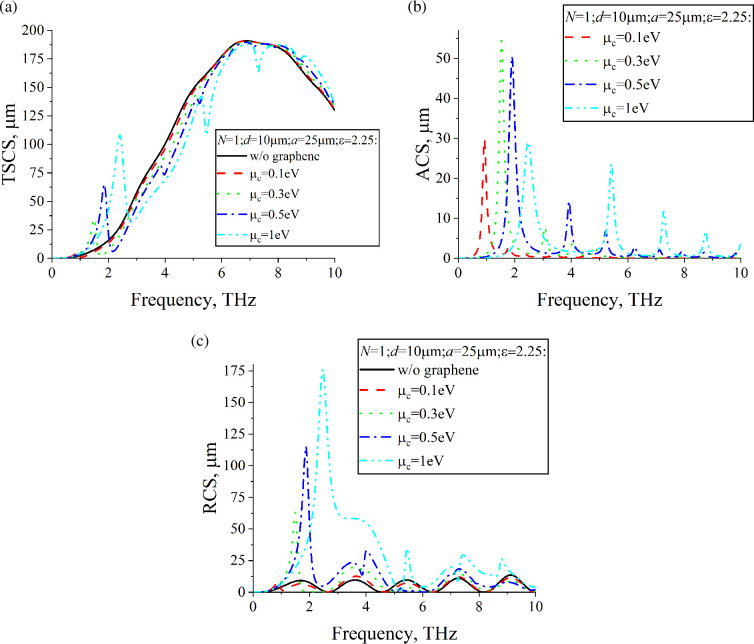
Dependences of (a) TSCS, (b) ACS and (c) RCS on the frequency for N=1, d=10µm, a=25µm, ε=2.25, $\phi_{0}=90^{0}$.

**Figure 5.**
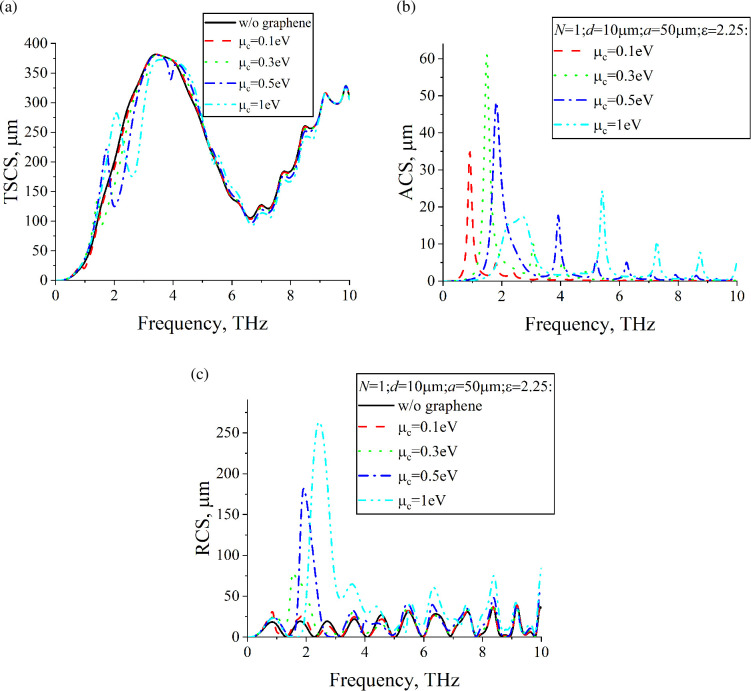
Same study as in figure 4, but for a=50µm.

**Figure 6.**
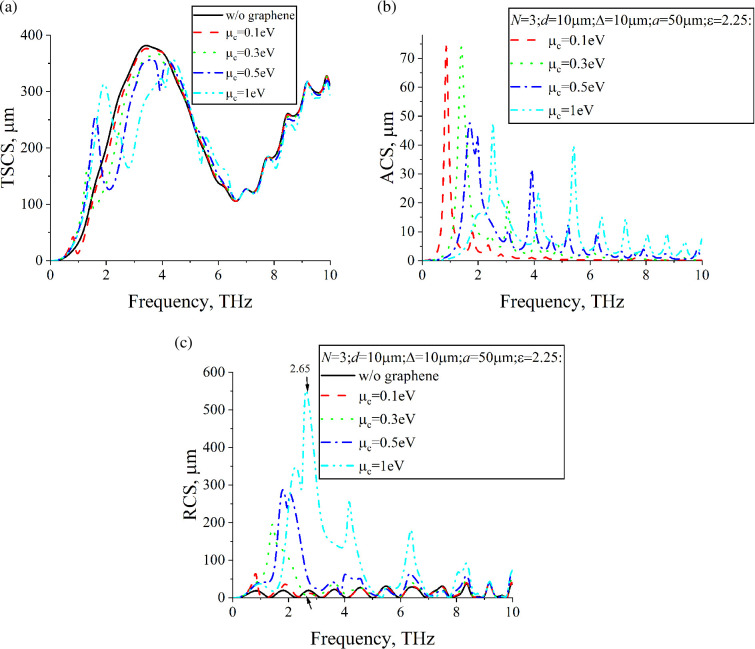
Same study as in figure 5, but for N=3, Δ=10µm.

Figure 4 shows dependences of TSCS, ACS and RCS of a lossless dielectric cylinder with a single, N=1, graphene strip on the frequency. The radius is *a* = 25 μm. For comparison, dependences for the dielectric cylinder without graphene strips are also presented. Figure 5 shows dependences of TSCS, ACS and RCS for a cylinder with twice the radius, *a* = 50 μm. However, for such a cylinder, it is possible to place N=3 strips of the same width as in figure 4 inside it. In figure 6, we represent dependences of TSCS, ACS and RCS for N=3 strips. Graphene parameters are τ=1 ps and room temperature T=300 K.

The frequency dependences of ACS allow for the identification of plasmon resonances. Since for figures 4– 6,6, the dielectric cylinder is lossless, the electromagnetic field is absorbed only by the graphene strips. The maxima of ACS correspond to the plasmon resonances. The first plasmon resonance is also noticeable in the dependences of TSCS. The scattering geometry consists of two sub-structures: the dielectric cylinder and the graphene strips. The frequency dependences of RCS demonstrate two families of maxima, each corresponding to one of these sub-structures. The RCS frequency dependences of the dielectric cylinder, in the absence of graphene strips, typically exhibit a periodic background. However, the insertion of graphene strips inside the cylinder results in prominent maxima near the plasmon resonance frequencies. The position of these plasmon resonances on the frequency axis can be controlled by variations in the chemical potential. At any frequency, the chemical potential of the graphene strips can be adjusted to achieve RCS values larger than or comparable to those of the bare dielectric cylinder. For certain parameter combinations, especially near the first plasmon resonance, adjusting the chemical potential can reduce the RCS to levels lower than those of the dielectric cylinder without graphene strips. In the case of several coplanar graphene strips, mutual coupling between them occurs, a phenomenon absent in the single-strip configuration. This coupling enhances the resonances, resulting in stronger scattering and absorption effects compared to a single strip. The presence of multiple strips also gives rise to super-modes, which introduce multiple maxima for each plasmon resonance, in contrast to the single-strip case, where only one maximum is observed per resonance. An increase in the number of graphene strips from *n* = 1 to *n* = 3 leads to a more than twofold increase in RCS for ${µ}_{c}=1$eV, with a resonance frequency shift of about 0.2 THz for the most pronounced maxima.

**Figure 7.**
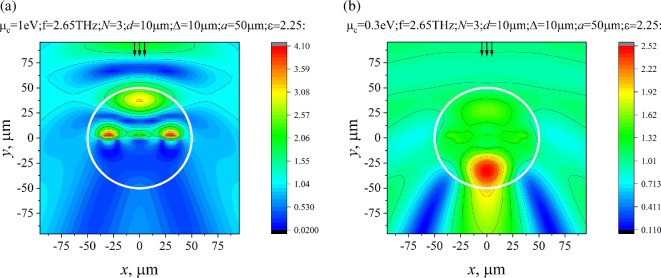
Near field, $\left|H_{z}\right|$, for f=2.65THz, N=3, d=10µm, Δ=10µm, a=50µm, ε=2.25, $\phi_{0}=90^{0}$. (a) ${µ}_{c}=1$ eV, (b) ${µ}_{c}=0.3$ eV. The border of the dielectric cylinder is shown as a white circle.

**Figure 8.**
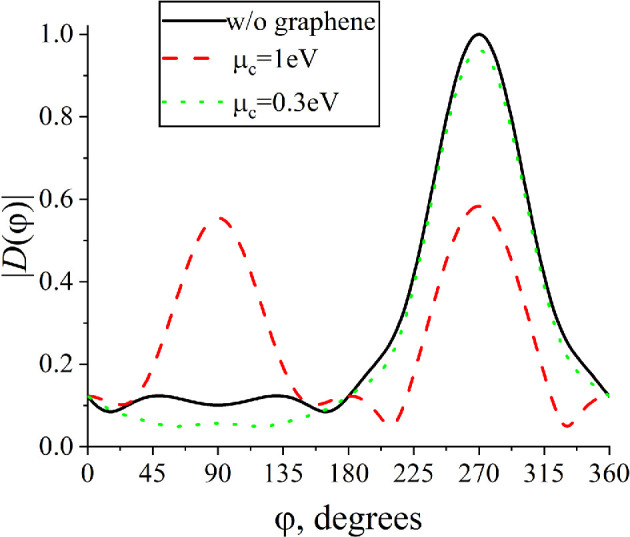
Normalized far-field radiation patterns (in amplitude) for f=2.65 THz, N=3, d=10µm, Δ=10µm, a=50µm, ε=2.25, $\phi_{0}=90^{0}$.

The near- and far-field patterns (in amplitude) are presented in figures 7 and 8 for the parameter values corresponding to the maximum and minimum of RCS (indicated by arrows in figure 6c). Near the first plasmon resonance (see figure 7a), the field distribution shows a clear maximum near the centre of the strips, whereas in figure 7b, the field maximum is shifted off the strips.

**Figure 9.**
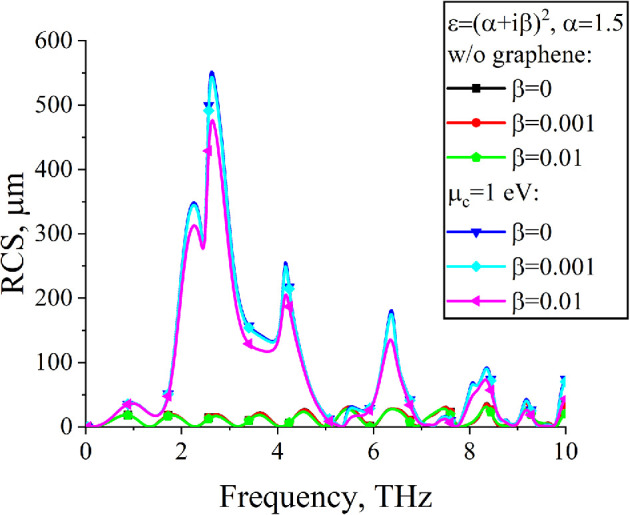
Dependences of RCS on the frequency for lossy dielectric, ${µ}_{c}=1$ eV, N=3, d=10µm, Δ=10µm, a=50µm, ε=2.25, $\phi_{0}=90^{0}$.

Figure 9 shows dependences of RCS for the dielectric with losses. The positions of the resonances on the frequency axis remain the same as in the case of a lossless dielectric. For small losses, the amplitude decreases insignificantly, indicating that plasmon resonances are robust against minor losses in the dielectric.

**Figure 10.**
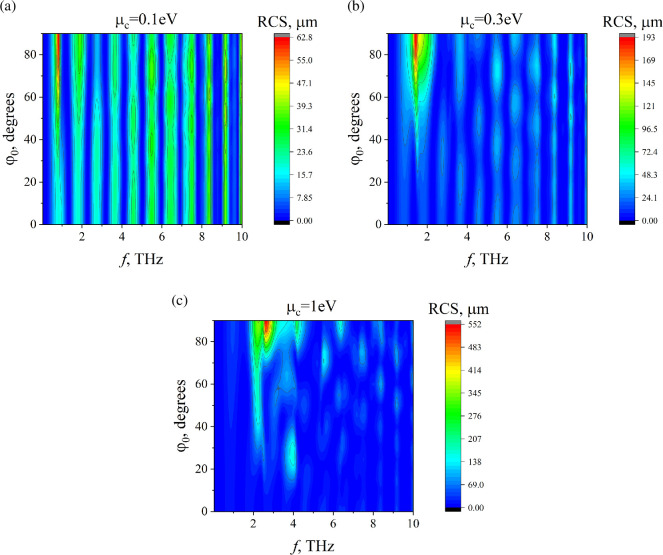
Dependences of RCS on the frequency and incidence angle for N=3, d=10µm, Δ=10µm, a=50µm, ε=2.25, $\phi_{0}=90^{0}$. (a) ${µ}_{c}=0.1$ eV, (b) ${µ}_{c}=0.3$ eV, (c) ${µ}_{c}=1$ eV.

To study the role of the incidence angle $\phi_{0}$, in figure 10, we plot colour maps of RCS as functions of two variables: the frequency f and the incidence angle $\phi_{0}$. The maps exhibit an almost periodic behaviour. In the case of normal incidence relative to the strip, $\phi_{0}=90^{0}$, only plasmon resonances with odd indices arise. However, for non-orthogonal incidence, plasmon resonances with even indices also appear, resulting in additional peaks for $\phi_{0}<90^{0}$. It is obvious that for $\phi_{0}=0^{0}$, the incident plane wave is not scattered by the graphene strips.

**Figure 11.**
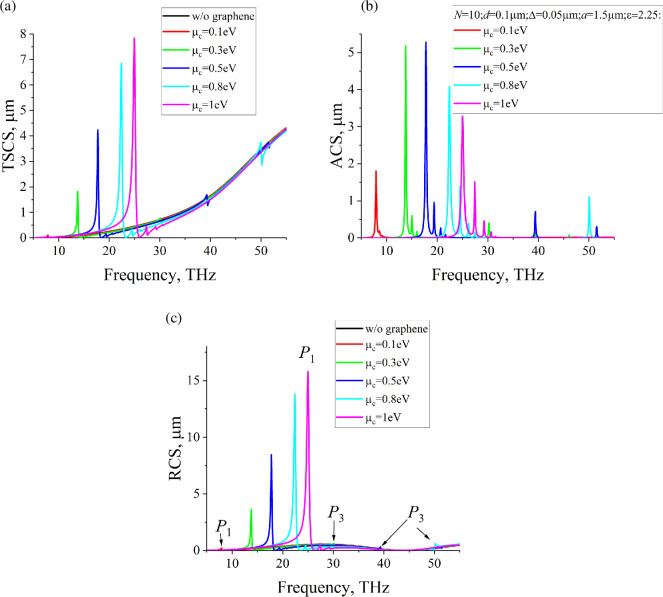
Dependences of (a) TSCS, (b) ACS, (c) RCS on the frequency for N=10, d=0.1µm, Δ=0.05µm, a=1.5µm, ε=2.25, $\phi_{0}=90^{0}$.

The results for the frequency band *f *= 5–55 THz are presented in figure 11. The radius of the dielectric cylinder is *a* = 1.5 μm. Taking into account the frequency dependence of the graphene conductivity σ, the frequencies of plasmon resonances get larger as $O\left(\sqrt{m/d}\right)$, where m is the number of resonance, if d→0. This behaviour contrasts with the resonances of the bare dielectric cylinder, where the frequencies scale as O(m/a). To ensure that plasmon resonances arise in the frequency band under consideration, we use a much smaller strip width compared with the radius, specifically d=0.1µm. For the chosen period l=0.25µm, we consider N=10 strips. The relatively large radius of the dielectric cylinder allows us to accommodate more graphene strips inside, enhancing the resonances. This configuration offers an advantage over models with a single strip, as the coupling between multiple strips leads to stronger and more tunable resonances, providing greater control over the system’s electromagnetic properties.

RCS exhibits much sharper peaks near the first plasmon resonances compared with figures 4–6. However, maxima near the high-order plasmon resonances are barely noticeable. For better visualization, we marked the first and third plasmon resonances as $P_{1}$ and $P_{3}$. The variation of the chemical potential also allows us to control the position of the plasmon resonances on the frequency axis, as well as the value of RCS. However, effective control of the RCS value is only possible in the frequency band near the first plasmon resonance.

## 5. Conclusions

We studied the scattering and absorption of the *H*-polarized plane wave by the dielectric cylinder with graphene strips inside it in a very wide frequency band from 0.1 to 55 THz. The solution, based on the hyper-singular boundary integral equations, is mathematically grounded. The Nystrom-type algorithm of discretization is full-wave and meshless. Our homemade code, besides controllable accuracy, is very fast.

We paid special attention to the modifications of RCS. We showed that RCS can be significantly increased, especially near the frequencies of the first plasmon resonance. The variation of the chemical potential allows us to control RCS over a wide range, making it possible to reduce it to a level lower than that for the dielectric cylinder without graphene strips. Additionally, the use of multiple coplanar strips enhances the resonances compared with a single strip, resulting in stronger scattering and absorption effects. The mutual coupling between the strips also leads to the formation of super-modes.

## Data Availability

The authors have presented the equations, which can be directly programmed by the reader, delivering all the results reported in the article.
